# Analysis of the Deflection, Bristle Splaying, and Abrasion of a Single Tuft of a Polybutylene Terephthalate Toothbrush after Use: A Randomized Controlled Trial

**DOI:** 10.3390/ma15144890

**Published:** 2022-07-14

**Authors:** Yoshino Kaneyasu, Hideo Shigeishi, Kouji Ohta, Masaru Sugiyama

**Affiliations:** Department of Public Oral Health, Program of Oral Health Sciences, Graduate School of Biomedical and Health Sciences, Hiroshima University, Hiroshima 734-8553, Japan; yoshi-kane@hiroshima-u.ac.jp (Y.K.); otkouji@hiroshima-u.ac.jp (K.O.); masaru@hiroshima-u.ac.jp (M.S.)

**Keywords:** toothbrush, polybutylene terephthalate, bristle deflection, randomized controlled trial

## Abstract

The aim of this study is to clarify the deflection, splaying, and abrasion of single tufts of polybutylene terephthalate (PBT) toothbrushes after use. A single-center randomized controlled trial is performed. The changes in deflection, bristle splaying, and abrasion are investigated for the middle single tuft of the top line (top–middle tuft) and the middle single tuft of the bottom line (bottom–middle tuft) of PBT toothbrushes with medium stiffness after 1 month, 2 months, and 3 months of use by 34 participants. A soft-material bending-resistance tester is used to assess the deflection of the single tufts. The deflection value of the top–middle tuft significantly increased after 1 month of use compared with the baseline. In contrast, the deflection of the bottom–middle tuft significantly increased after 3 months of use compared with the baseline and after 1 month and 2 months of use. Importantly, the change in deflection was distinctly different between the top– and bottom–middle tufts. The bristle splaying of both tufts significantly increased after use, but a significant change in bristle abrasion was not found. The bending stiffness of the top tuft of a PBT toothbrush may decrease more rapidly than that of the bottom tuft with use.

## 1. Introduction

To perform effective toothbrushing for dental plaque removal, it is important to know how rapidly a toothbrush deteriorates with use. It is thought that toothbrush deterioration (i.e., bristle wear and abrasion and the decrease in bristle stiffness), as well as toothbrushing technique affect the removal of dental plaque [1,2]. We have previously reported that polybutylene terephthalate (PBT) manual toothbrushes become less efficient for plaque removal owing to increasing bristle splaying after 2 months of use [3]. PBT is a polyester-based thermoplastic material with low moisture absorption [4], and it is therefore used for wet applications, such as toothbrushes. Additionally, the results of the International Organization for Standardization (ISO) 22254 test [5] have revealed that the bristle stiffness of PBT toothbrushes with soft stiffness and medium stiffness significantly decreases after 2 months of use [6]. These results suggest that a PBT toothbrush should be replaced approximately every 2 months because of deteriorating bristles, as well as decreased plaque removal efficacy. However, the changes in bristle deflection and splaying in each tuft of a PBT toothbrush with use remain unknown. We hypothesize that the changes in bristle deflection and the splaying of different tufts may vary with use.

The methods used to investigate the mechanical properties of synthetic polymers include tensile testing according to ISO 527, the flexural strength test, and the stiffness test [7,8,9]. The bristle diameter and length, as well as the bristle material, can affect the bristle stiffness of a toothbrush [10]. Methods for evaluating the bristle stiffness include methods for measuring the tuft retention strength and a mathematical model for predicting stiffness [5,11,12,13,14]. Rawls et al. [14] reported that there is a discrepancy in the values of bristle stiffness between the measurement and calculation methods. The bristle stiffness is commonly evaluated in accordance with the compressive strength test based on Japanese Industrial Standards (JIS S3016) [11]. In contrast, the ISO 22254 test is widely used to determine the resistance of the tufted portion of a manual toothbrush to deflection [5]. However, the ISO 22254 test is not applicable to measure the deflection of each tuft of a toothbrush. A micro-hardness tester is commonly used to accurately measure the stiffness of hard and soft materials [15,16]. It is thought that a soft-material bending-resistance tester can be used to measure the bending stiffness of a single tuft. Therefore, in this study, a soft-material bending-resistance tester is used to measure the deflection of a single tuft of PBT toothbrushes.

The changes in the physical properties of a single tuft of a PBT toothbrush with use have not been fully elucidated. Therefore, in this study, we investigate the changes in the deflection and splaying of single tufts of PBT toothbrushes with medium stiffness after 1 month, 2 months, and 3 months of use. In addition, the change in bristle abrasion is evaluated using a scanning electron microscope (SEM).

## 2. Materials and Methods

### 2.1. Study Design

A single-center randomized controlled trial (RCT) was conducted to evaluate the changes in the bristle stiffness and bristle splaying of PBT toothbrushes with use [3]. A total of 80 people who met the eligibility criteria for this study were recruited from November 2016 to September 2017. The study design was approved by the Ethical Committee of Hiroshima University (title: Randomized controlled trial on the efficiency in removal of dental plaque related to changes of bristles’ hardness of toothbrushes, No. C-120), and all of the participants signed informed consent agreements. The inclusion and exclusion criteria and random allocation of the participants have been explained in our previous paper [3]. The participants were randomly assigned to two groups, and a toothbrush with soft stiffness (i.e., Tuft 24 Soft) or a toothbrush with medium stiffness (i.e., Tuft 24 Medium) was allocated to each of the 40 participants in the groups.

To minimize the individual differences in the toothbrushing method, all of the participants received toothbrushing instructions on the scrubbing method by a dental hygienist before starting the study. The participants were instructed to hold the brush with a pencil grip and brush their teeth gently with a horizontal and small scrubbing motion. Additionally, the toothbrushing pressure was checked using a toothbrushing pressure measuring device (Comatsu Co., Ltd., Saitama, Japan) before and after starting the study to prevent toothbrushing with too much pressure. The participants performed toothbrushing for 3 min twice a day with toothpaste covering half of the toothbrush surface. Additionally, the participants stored the toothbrushes at room temperature during participation. Each toothbrush was collected after the first use (M0), after 1 month of use (M1), after 2 months of use (M2), and after 3 months of use (M3). Six participants in the medium toothbrush group and two participants in the soft toothbrush group discontinued participation for personal reasons. In this study, bristle deflection, splaying, and abrasion were investigated for single tufts of PBT toothbrushes with medium stiffness for 34 participants as the first step.

### 2.2. Measurement of Deflection of a Single Tuft

**Figure 1:**
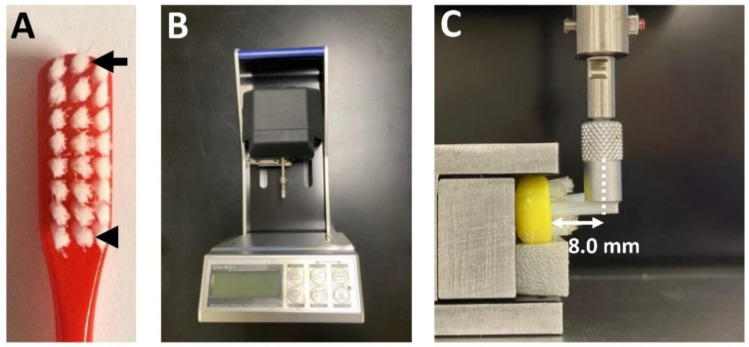
Measurement of the deflection value of the top– and bottom–middle tufts using a bending resistance tester. (**A**) Top– (arrow) and bottom–middle (arrowhead) tufts of the PBT toothbrush. (**B**) Bending-resistance tester. (**C**) Measurement of deflection of a single tuft using the bending-resistance tester.

The medium toothbrush used in this study contained approximately 40 monofilaments (monofilament length 9.0 mm, monofilament diameter 0.2 mm) in each tuft. The deflection of a middle single tuft at the top line (i.e., top–middle tuft) and a middle single tuft at the bottom line (i.e., bottom–middle tuft) were evaluated (Figure 1A). To measure the deflection of the two tufts, the top–middle tuft and bottom–middle tuft remained and the other tufts were cut from the toothbrush head. Because the single tufts needed to be isolated as much as possible from the other tufts to measure the deflection of the single tuft alone, we chose to investigate the top–middle tuft and bottom–middle tuft. The deflection of the top– and bottom–middle tufts was measured with a soft-material bending-resistance tester (Micro-Measuring Force Hardness Tester, CH-R01/IRHD, CITIZEN Seimitsu, Yamanashi, Japan) (Figure 1B) in accordance with the manufacturer’s instructions. This bending resistance test was based on the measurement of the indentation depth. The toothbrush head was stored in water at 37 ± 2 °C for 90 s according to ISO 22254 before the deflection of the single tufts was measured. The end of the measurement terminal (flat type, 4.0 mm diameter) was vertically placed on a single tuft 8.0 mm from the base of the toothbrush head (Figure 1C). The indentation depth of the measurement terminal was measured five times. The average indentation depth (mm) and load value (5 mN) were used to calculate the deflection value. The deflection value was calculated by the following equation: deflection value (mm/mN) = average indentation depth (mm)/5 mN.

### 2.3. Measurement of Bristle Splaying Using Digital Software

**Figure 2:**
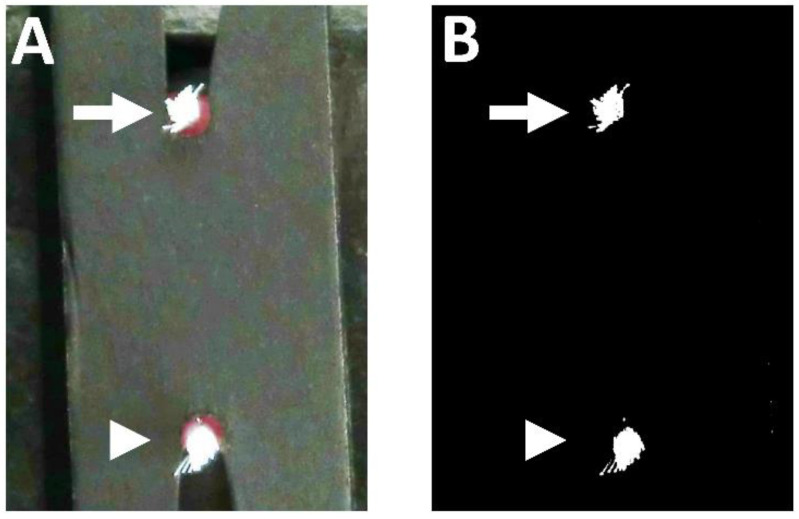
Digital photograph of the toothbrush head. (**A**) Toothbrush head shielded by black paper. Top– (arrow) and bottom–middle (arrowhead) tufts of the PBT toothbrush. (**B**) Black and white images of the toothbrush head. Top– (arrow) and bottom–middle (arrowhead) tufts of the PBT toothbrush.

The measurement method of bristle splaying using a photo-taking set and digital software is described in our previous paper [3]. The head of the toothbrush was shielded by black paper to prevent light reflection from the toothbrush head (Figure 2A). A photograph of the front view of the toothbrush head was taken by the photo-taking set with a digital camera [3]. After preparing black and white pictures of the tuft bristle (Figure 2B), the white region was digitally analyzed using NIH ImageJ software.

### 2.4. Evaluation of Bristle Abrasion Using a SEM

**Figure 3:**
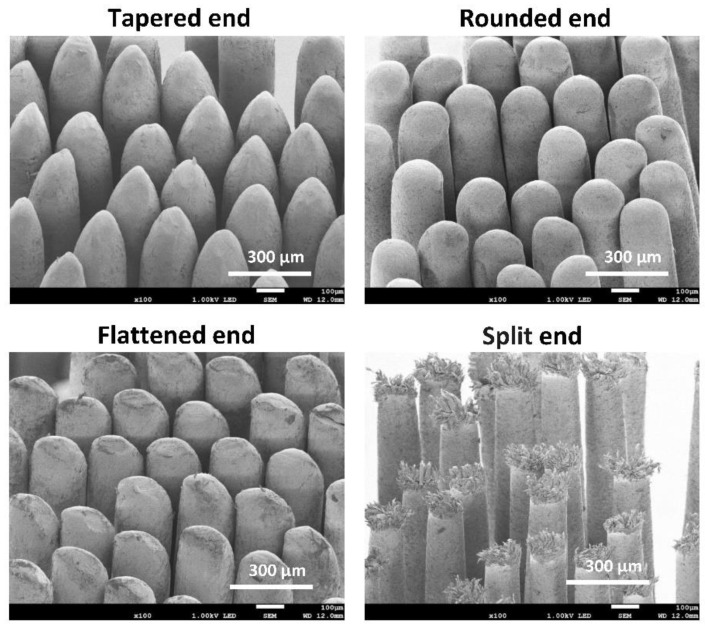
SEM images of the bristle end. A bristle with a tapered end, a rounded end, a flattened end, and a split end.

A SEM (JSM-7200F, JEOL Ltd., Tokyo, Japan) was used to examine bristle abrasion. The bristle surface was examined by a SEM at 1.0 kV accelerating voltage at 30× and 100× magnification after a gold coating was applied using a sputtering device (JFC-3000FC, JEOL Ltd.) [17]. The two-dimensional SEM images obtained from directly above and at 70° to the bristle surface were used with a working distance of 12 mm. A bristle was scored in accordance with the shape of the bristle end in the SEM image. A bristle with a tapered shape or a rounded end was considered to be acceptable and scored 1 (Figure 3). A bristle with a flattened end was considered to show clear signs of wear and scored 2 (Figure 3). A bristle with a split end was considered to show severe signs of wear and scored 3 (Figure 3). The bristle abrasion score per tuft was calculated as the total abrasion score divided by the total number of bristles of the tuft (i.e., bristle abrasion score = total abrasion score per tuft/total number of bristles per tuft).

### 2.5. Statistical Analysis

The results were statistically analyzed using JMP Pro software (version 15.0.0, SAS Institute Inc., Cary, NC, USA). The Mann–Whitney *U* test was used to compare the differences between the two groups. The Steel–Dwass test was used for multiple comparisons. *p* < 0.05 was considered to indicate statistical significance.

## 3. Results

### 3.1. Changes in the Deflection of the Top– and Bottom–Middle Tufts of the PBT Toothbrushes

**Figure 4:**
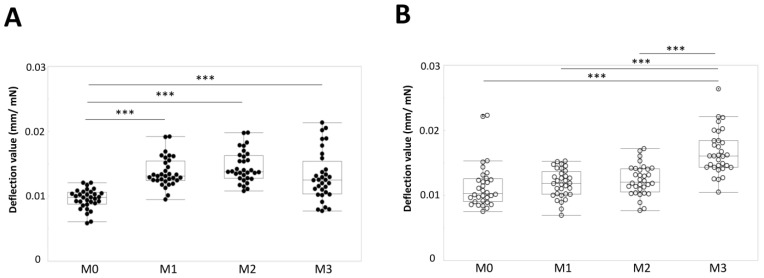
Deflection values of the top– and bottom–middle tufts at M0, M1, M2, and M3. (**A**) Top–middle tuft. *** *p* < 0.001. (**B**) Bottom–middle tuft. *** *p* < 0.001.

The deflection values of the top– and bottom–middle tufts were measured at M0, M1, M2, and M3. The deflection value of the top–middle tuft significantly increased at M1, M2, and M3 compared with M0 (*p* < 0.001, *p* < 0.001, and *p* < 0.001, respectively, Steel–Dwass test) (Figure 4A). No significant difference of deflection was found between M1 and M2, M1 and M3, or M2 and M3 (Figure 4A). In contrast, a significant increase in the deflection value of the bottom–middle tuft was not found at M1 and M2 compared with M0 (Figure 4B). The deflection value of the bottom–middle tuft significantly increased at M3 compared with M0, M1, and M2 (*p* < 0.001, *p* < 0.001, and *p* < 0.001, respectively, Steel–Dwass test) (Figure 4B). In addition, a significant difference in the deflection value was found between the top– and bottom–middle tufts at M0, M1, M2, and M3 (Mann–Whitney *U* test) (Table 1). These results suggest that the bending stiffness of the bottom–middle tuft can be maintained for 2 months, but rapidly decreases after 3 months. Importantly, the change in deflection was distinctly different between the top– and bottom–middle tufts.

### 3.2. Changes in Bristle Splaying of the Top– and Bottom–Middle Tufts of the PBT Toothbrushes

**Figure 5:**
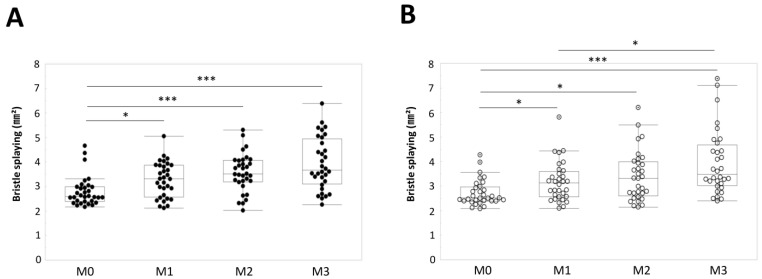
Bristle splaying of the top– and bottom–middle tufts at M0, M1, M2, and M3. (**A**) Top–middle tuft. * *p* < 0.05. *** *p* < 0.001. (**B**) Bottom–middle tuft. * *p* < 0.05. *** *p* < 0.001.

Bristle splaying of the top– and bottom–middle tufts of the medium PBT toothbrushes was measured at M0, M1, M2, and M3. The mean bristle splaying of the top–middle tuft significantly increased at M1, M2, and M3 compared with at M0 (*p* < 0.05, *p* < 0.001, and *p* < 0.001, respectively, Steel–Dwass test) (Figure 5A). The mean bristle splaying of the bottom–middle tuft significantly increased at M1, M2, and M3 compared with at M0 (*p* < 0.05, *p* < 0.05, and *p* < 0.001, respectively, Steel–Dwass test) (Figure 5B). A significant increase in bristle splaying was found for both tufts after 1 month of use. No significant difference in bristle splaying was found between the top– and bottom–middle tufts at M0, M1, M2, and M3 (Table 2).

### 3.3. Changes in Bristle Abrasion of the Top– and Bottom–Middle Tufts of the PBT Toothbrushes

**Figure 6:**
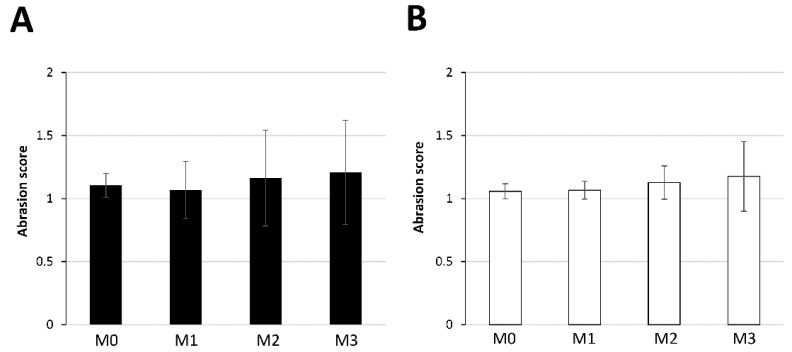
Bristle abrasion scores of the top– and bottom–middle tufts at M0, M1, M2, and M3. (**A**) Top–middle tuft. (**B**) Bottom–middle tuft.

Bristle abrasion of the top– and bottom–middle tufts of the PBT toothbrushes was investigated at M0, M1, M2, and M3. The abrasion scores are shown in Figure 6. The bristle abrasion scores of both tufts slightly increased after use, but there was no significant difference in either tuft (Figure 6). There was no significant difference in bristle abrasion between the top– and bottom–middle tufts at M0, M1, M2, or M3 (Table 3).

## 4. Discussion

ISO has specified a test method for measuring the resistance to deflection of the tufted portion of a conventional manual toothbrush [5]. In a previous study, we assessed the resistance to deflection of a PBT toothbrush in accordance with the ISO 22254 test method [6]. In this study, we found that the deflection of a single tuft of a PBT toothbrush can be measured using a soft-material bending-resistance tester. To our knowledge, this is the first report of measuring the stiffness of each tuft of a toothbrush using a soft-material bending-resistance tester. A soft-material bending-resistance tester enables the evaluation of the resistance of a single tuft of a toothbrush to deflection.

The bristle stiffness of a toothbrush is associated with safe and effective toothbrushing [18,19]. In a recent study, we found that the bristle stiffness of a PBT toothbrush significantly decreased 2 months after use [6]. The bristle stiffness of a PBT toothbrush gradually decreased with use [6]. In this study, the deflection of the top–middle tuft increased more rapidly compared with that of the bottom–middle tuft for a PBT toothbrush with medium stiffness. It is likely that the bending stiffness of the bottom–middle tuft was maintained until 2 months of use. The participants performed regular toothbrushing, and the toothbrushing pressure was regularly checked during participation in the study. Therefore, it is likely that the individual differences in toothbrushing had little effect on the results of this study. The results suggest that the change in the bending stiffness of a single tuft depends on the site of the tuft. It is speculated that the bottom–middle tuft may be less susceptible to the mechanical impact of toothbrushing compared with the top–middle tuft until 2 months of use. The top–middle tuft may be more susceptible to mechanical impact during a short period after use. However, the reason why the deflection of the top–middle tuft did not change after 2 and 3 months of use compared with after 1 month of use remains unknown. One possible reason is a tuft other than the top tuft (e.g., a single tuft at the middle or bottom line) may alternatively become susceptible to mechanical impact after 2 months of use. Overall, the change in the bending stiffness of a single tuft seems to be different depending on the location of the tuft on the PBT toothbrush. An additional study is required to investigate the decreases in the bending stiffness of different tufts because we could not simultaneously investigate several tufts. Furthermore, the bending stiffness changes in different tufts of PBT toothbrushes with soft stiffness remain unknown. Therefore, a further study is necessary to clarify whether similar results are obtained for a single tuft of a PBT toothbrush with soft stiffness.

We speculate that the deflection value of the top–middle tuft at M3 was greater than at M1 and M2. However, the mean deflection value of the top–middle tufts at M3 was smaller than at M1 and M2, but the difference was not significant. In this study, each toothbrush was evaluated independently after 1 month of use, after 2 months of use, and after 3 months of use. Thus, the toothbrushes collected after 3 months of use were not the same toothbrushes as the toothbrushes collected after 1 month of use or 2 months of use. Therefore, the small decrease in the deflection value at M3 can be attributed to the independent examination of the toothbrushes in this study. Bristle splaying increased with time for both tufts. However, the deflection of the bottom–middle tuft did not significantly change until 2 months of use. In addition, no significant change in the deflection of the top–middle tuft was found after 2 months of use. These results suggest that bristle splaying may not be correlated with the bending stiffness of the top– and bottom–middle tufts of PBT toothbrushes.

The bristle ends of a toothbrush play an important role in dental plaque removal by directly reaching the tooth surface. The worn shape of bristle ends can cause incidences of gingival abrasion [20]. This highlights the importance of regularly changing a toothbrush with sharpened bristle tips for a new toothbrush to prevent gingival abrasion. The abrasion of the nylon bristles of different types of toothbrushes has been accurately investigated using a microscope or a SEM [21,22,23,24,25,26,27,28]. The round end of a nylon bristle changes to a sharp-edged end, a flattened end, or a severely damaged end (e.g., cracking and splitting of the bristle material) with use [21,22,23]. In contrast, the abrasion of PBT bristles with use has not been fully investigated. In this study, the tapered end of the PBT bristle changed to a flattened end with use, but a severely damaged end (i.e., a split end) was rarely found. A significant increase in the bristle abrasion score was not found after use, indicating that PBT bristles may be less abrasive than nylon bristles. Bristle abrasion is thought to be associated with the quality of the material (e.g., *polyester* or nylon), as well as the brushing force and toothbrushing frequency [23,29]. However, from this study, it remains unknown whether PBT bristles are less wearing than nylon bristles.

PBT toothbrushes have been commonly used in Japan in recent years [30]. It is speculated that PBT toothbrushes rather than nylon toothbrushes may be more commonly and widely used in the future because of several merits of PBT, such as low moisture absorption, heat resistance, and chemical resistance [4]. We believe that our results will contribute to the research of PBT materials to some degree. To the best of our knowledge, this is the first study to investigate the changes in bristle deflection and the splaying of tufts of PBT toothbrushes.

There are some limitations to this study. The bending stiffness of the top–middle tuft was more rapidly downregulated compared with the bottom–middle tuft. This may result in reduction in the plaque removal efficiency. However, it remains unclear whether the change in the top tufts affects the plaque removal efficiency. Additional investigation is required to determine the effect of the decrease in the bending resistance of the top tufts on plaque removal. We preliminary investigated the bristle deflection and splaying of two representative tufts (i.e., the top and bottom tufts) in this study. However, the changes in the bristle deflection and splaying of the middle tuft remain unknown. Additionally, it remains unknown how the bristle stiffness and splaying change in tufts made from other materials (i.e., polyester and nylon) with use. However, it is speculated that the top tuft is more susceptible to mechanical stress by brushing compared with other tufts, regardless of the type of bristle material. Therefore, the use of more durable bristle materials for the top tuft of toothbrushes should be considered.

## 5. Conclusions

The changes in the bending stiffness of single tufts of PBT toothbrushes with medium stiffness differed depending on the location of the tufts. The bending stiffness of the top tuft decreased with use more rapidly than that of the bottom tuft. A soft-material bending-resistance tester is applicable to evaluate the resistance of a single tuft of a PBT toothbrush to deflection. The bristle splaying of both tufts significantly increased with use. A further study is necessary to clarify whether the changes in bending stiffness with use are different for each tuft.

## Figures and Tables

**Table 1:** Deflection values of the top– and bottom–middle tufts of the PBT toothbrushes.

	Top Tuft	Bottom Tuft	
	Mean ± SD (mm/mN)	Mean ± SD (mm/mN)	*p*-Value ^†^
M0	0.010 ± 0.002	0.011 ± 0.003	<0.05
M1	0.014 ± 0.002	0.012 ± 0.002	<0.01
M2	0.015 ± 0.002	0.012 ± 0.002	<0.01
M3	0.013 ± 0.004	0.017 ± 0.003	<0.001

^†^ Mann–Whitney *U* test. *p*-values less than 0.05 were considered statistically significant.

**Table 2:** Bristle splaying of the top– and bottom–middle tufts of the PBT toothbrushes.

	Top Tuft	Bottom Tuft	
	Mean ± SD (mm^2^)	Mean ± SD (mm^2^)	*p*-Value ^†^
M0	2.80 ± 0.60	2.72 ± 0.52	N.S.
M1	3.28 ± 0.72	3.18 ± 0.81	N.S.
M2	3.56 ± 0.78	3.41 ± 1.01	N.S.
M3	3.94 ± 1.07	3.95 ± 1.31	N.S.

^†^ Mann–Whitney *U* test. N.S.: not significant.

**Table 3:** Bristle abrasion scores of the top– and bottom–middle tufts of the PBT toothbrushes.

	Top Tuft	Bottom Tuft	
	Mean ± SD	Mean ± SD	*p*-Value ^†^
M0	1.10 ± 0.09	1.06 ± 0.06	N.S.
M1	1.07 ± 0.23	1.07 ± 0.07	N.S.
M2	1.16 ± 0.38	1.13 ± 0.13	N.S.
M3	1.21 ± 0.41	1.18 ± 0.28	N.S.

^†^ Mann–Whitney *U* test. N.S.: not significant.

## Data Availability

All data generated or analyzed in this study are included in this article.
